# Lectin from *Canavalia brasiliensis* Seeds (ConBr) Is a Valuable Biotechnological Tool to Stimulate the Growth of *Rhizobium tropici in Vitro*

**DOI:** 10.3390/molecules17055244

**Published:** 2012-05-07

**Authors:** Mayron Alves de Vasconcelos, Claudio Oliveira Cunha, Francisco Vassiliepe Sousa Arruda, Victor Alves Carneiro, Fabio Martins Mercante, Luiz Gonzaga do Nascimento Neto, Giselly Soares de Sousa, Bruno Anderson Matias Rocha, Edson Holanda Teixeira, Benildo Sousa Cavada, Ricardo Pires dos Santos

**Affiliations:** 1 Department of Biochemistry and Molecular Biology, Federal University of Ceara, Fortaleza, CE 60440-970, Brazil; Email: mayronvasconcelos@gmail.com (M.A.V.); agrobrasil@gmail.com (C.O.C.); brunoanderson@gmail.com (B.A.M.R.); 2 Integrated Laboratory of Biomolecules (LIBS), School of Medicine, Federal University of Ceara, Sobral, CE 62042-280, Brazil; Email: vassiliepe@gmail.com (F.V.S.A.); victorcarneiro@ufc.br (V.A.C.); ziullec@gmail.com (L.G.N.N.); edsonlec@gmail.com (E.H.T.); 3 Embrapa Western Region Agriculture, Dourados, MS 79804-970, Brazil; Email: mercante@cpao.embrapa.br; 4 Computer Engineering/Biotechnology Center of Sobral, Federal University of Ceara, CE 62011-000, Brazil; Email: giselly.eng@gmail.com

**Keywords:** lectin, *Canavalia brasiliensis*, *Canavalia ensiformis*, *Rhizobium tropici*, interaction, growth

## Abstract

To study the interactions between a *Rhizobium tropici* strain and lectins isolated from the seeds of *Canavalia ensiformis* (ConA) and *Canavalia brasiliensis* (ConBr), a lectin fluorescence assay was performed. In addition, an experiment was designed to evaluate the effect of the two lectins on bacterial growth. Both lectins were found to bind to *R. tropici* cells, but the interactions were inhibited by D-mannose. Interestingly, only ConBr stimulated bacterial growth in proportion to the concentrations used (15.6–500 µg/mL), and the bacterial growth stimulation was inhibited by D-mannose as well. Structure/Function analyses by bioinformatics were carried out to evaluate the volume and carbohydrate recognition domain (CRD) configuration of ConA and ConBr. The difference of spatial arrangement and volume of CRD may indicate the variation between biological activities of both lectins. The results suggest that ConBr could be a promising tool for studies focusing on the interactions between rhizobia and host plants.

## 1. Introduction

Diazotrophic bacteria capable of forming symbiotic N_2_-fixing associations with many plant species of the Leguminosae family are collectively known as rhizobia [1]. The establishment of symbiosis between rhizobia and host plants requires the recognition and exchange of molecular signals [2]. Several signaling molecules are exchanged between plant and bacterium (such as flavonoids, betaines and aldonic acid), thereby regulating the initiation, differentiation and functioning of the nodule [3].

*Rhizobium tropici*, a major symbiont of the common bean (*Phaseolus vulgaris*) in tropical soils, is abundant in all Brazilian biomes [4]. Certain strains of *R. tropici* are particularly effective and competitive at nitrogen fixation [5,6].

Lectins recognize and bind reversibly to carbohydrate epitopes with high specificity. They have a number of different functions in nature, the most important of which is acting as plant defense proteins and information mediators in biological systems through interactions with glycoproteins, glycolipids and oligosaccharides. Lectins can interact with bacterial polysaccharides, mainly lipopolysaccharides (LPS), to promote bacterial adhesion and improve N_2_ fixation [7,8]. Lectins also stimulate cellular respiration, induce bacterial growth and modulate metabolism [9,10,11,12]. The lectins ConA and ConBr were isolated from the seeds of two species of the Diocleinae subtribe, *Canavalia ensiformis* and *Canavalia brasiliensis*, respectively. Although lectins isolated from Diocleinae seeds have a high degree of similarity and bind to glucose and mannose [13], they differ in their biological activity [14,15,16].

The objective of this study was to investigate the interaction between *Rhizobium tropici* CIAT 899 and the homologous lectins ConA and ConBr and to evaluate the effect of these proteins on bacterial growth.

## 2. Results and Discussion

**Figure 1:**
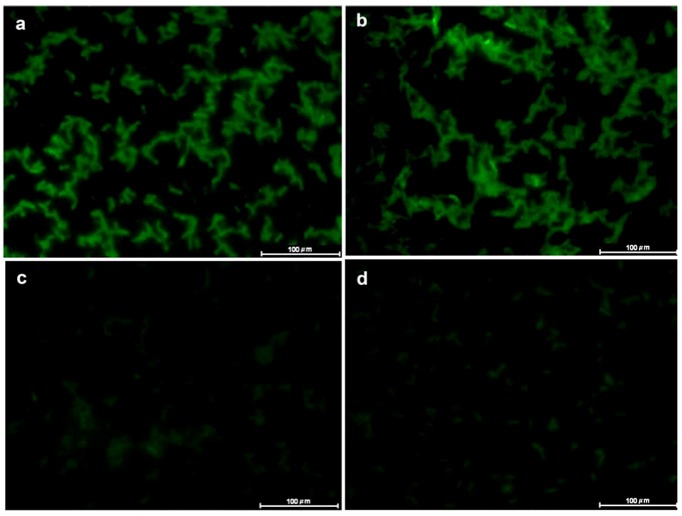
Lectin-fluorescence microscopy of interaction between FITC-lectins and *Rhizobium tropici* CIAT 899. (**a**) FITC-ConA; (**b**) FITC-ConBr; (**c**) FITC-ConA plus D-mannose; and (**d**) FITC-ConBr plus D-mannose.

Using fluorescence assays, this study showed that both ConA and ConBr interacted with *Rhizobium tropici*. The two lectins had similar fluorescence emission profiles (Figure 1a,b). When D-mannose was added to ConA and ConBr solutions, interaction between the lectin and bacterial surface decreased abruptly (Figure 1c,d). No fluorescence was observed when the cells were treated with FITC-BSA or FITC alone. Likewise, no intrinsic fluorescence was detected (data not shown).

**Figure 2:**
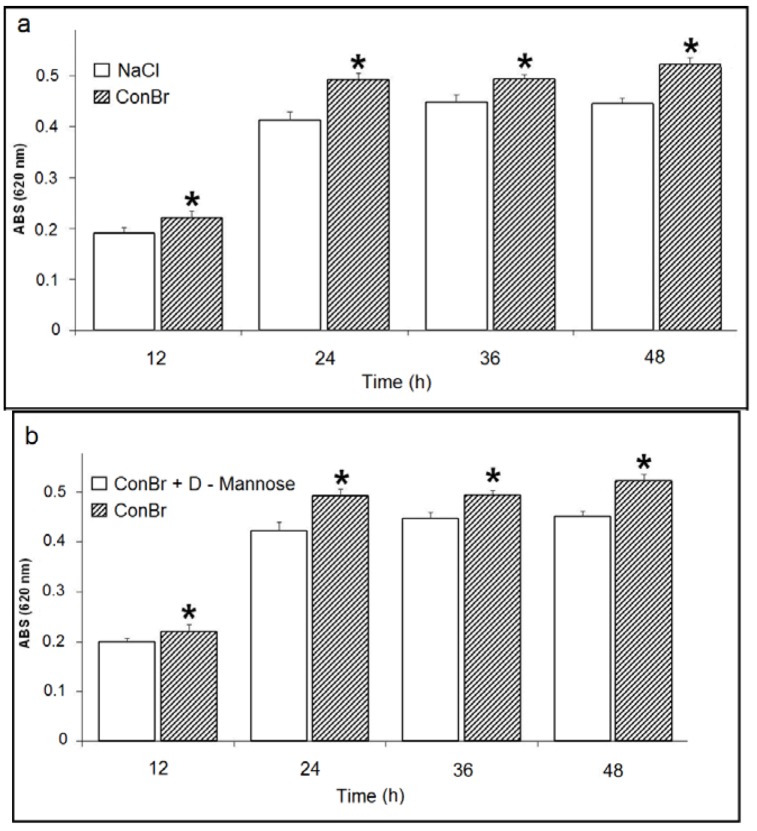
(**a**) Effect of ConBr on the growth of *R. tropici*. (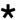) *p* *<* 0.01 related to 0.15 M NaCl; (**b**) Comparison between the effects of ConBr and ConBr complexed with D-mannose on the growth of *R. tropici*. (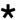) *p* *<* 0.01 related to ConBr plus D-mannose.

As shown in the Figure 2a, ConBr had a stimulatory effect on the growth of *R. tropici* (500 µg/mL), as compared to NaCl control. This effect was observed for all experimental incubation periods (12, 24, 36 and 48 h). Similarly, ConBr enhanced bacterial growth as compared to the ConBr-D-mannose complex (Figure 2b).

**Figure 3:**
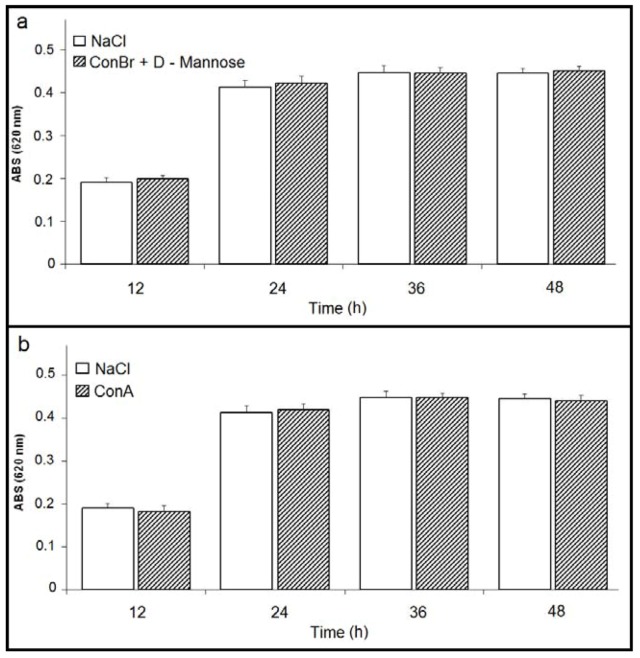
(**a**) Effect of ConBr complexed with D-mannose on the growth of *R. tropici*; (**b**) Effect of ConA on the growth of *R. tropici*.

Nevertheless, when ConBr was inhibited by the addition of D-mannose, no significant change in bacterial growth was reported (Figure 3a). Both ConA (Figure 3b) and ConA complexed with D-mannose had no effects on the growth of *R. tropici* (data not shown), similar to the 0.1 M D-mannose negative control (data not shown).

Lectins can recognize and bind reversibly to carbohydrates on the cell surface and interact with cell wall polysaccharides and/or glycoconjugates in the cell membrane [17,18]. Carbohydrates are directly involved in many biological processes, both physiological and pathological [18,19]. Strathmann and colleagues [20] showed that ConA isolated from seeds of *Canavalia ensiformis* interacted with carbohydrates present in the extracellular matrix of the Gram-negative bacterium *Pseudomonas aeruginosa*. Likewise, another glucose/mannose-specific lectin isolated from the roots of *Sesbania aculeate* interacted with different *Rhizobium* strains [21].

Lectins are known to interact with nitrogen-fixing bacteria and play an important role in metabolism. For example, wheat germ agglutinin (WGA), is a *N*-acetylglucosamine-binding lectin isolated from wheat germ, stimulated N_2_ fixing, ammonia excretion, glutamine synthetase activity and indole acetic acid production in *Azospirillum brasiliensis *[22]. In addition, *Vatairea macrocarpa* lectin (VML) enhanced H^+^ efflux in *Rhizobium tropici* [10]. Previously, Martinez and colleagues [11] demonstrated that VML and ConBr stimulated the respiration of *R. tropici* and *Rhizobium etli*.

In the present study, ConBr bound to *Rhizobium tropici* and stimulated bacterial growth probably by modifying its metabolism. However, the effect was neutralized by the presence of its specific sugar D-mannose. These findings were consistent with those published by Bajaj and colleagues [9] who demonstrated that the D-glucose-specific lectin of *Pisum sativum* stimulated the growth of *Rhizobium leguminosarum*, probably by enhancing nutrient transport.

Although ConA also binds to bacteria, it displayed no discernible activity in experiments. Similar results were reported by Araújo-Filho and colleagues [23] for three Diocleinae lectins: *Canavalia ensiformis* (ConA), *Canavalia maritima* (ConM) and *Dioclea guianensis* (Dgui). All lectins were found to bind spores of *Colletotrichum gloeosporioides*, but only Dgui inhibited germination.

Although Diocleinae lectins are similar in many respects, they vary significantly with regard to biological activity [13,24,25]. In this study, the growth of *Rhizobium tropici* was stimulated *in vitro* by the glucose/mannose-binding lectin ConBr, but not the homologous lectin ConA. Differences in biological activity between homologous lectins may be due to small changes in the configuration of important residues in the carbohydrate recognition domain (CRD) [26] or to pH-dependent oligomerization [13].

Structural comparisons showed that ConA and ConBr differ by only four residues: Asp58, Ala70, Asp151 and Glu155 in ConA *versus* Gly58, Gly70, Glu151 and Arg155 in ConBr (PDB codes: 1AZD and 1JBC). The CRDs of Diocleinae lectins, such as ConA and ConBr, consist of highly conserved residues (Tyr12, Asn14, Leu99, Tyr100, Asp208 and Arg228). These residues are key elements in the interaction between lectin and carbohydrate [27]. The difference in biological activity between ConA and ConBr has been attributed to a non-conservative substitution (amino acid in position 58), which makes the CRD more open in ConBr [28]. This more open conformation was due to the CRD design that induces a reduction in the volume of the ConBr carbohydrate binding site [29]. To show that highly similar Diocleinae lectins can nevertheless interact differently with carbohydrates, Ramos and colleagues [30] evaluated the fine glycan specificity of Diocleinae lectins for a number of glycoproteins using surface plasmon resonance. ConBr was observed to be much more reactive than ConA or ConM, suggesting that the carbohydrate binding site might be extended by neighboring surface residues [30]. Moreover, ConA displays homogenous behavior above pH 7.0, whereas ConBr presents a mixture of dimers (31%) and tetramers (69%) at pH 8.0 [28]. According to Cavada and colleagues [13], differences in the proportions of tetrameric and dimeric forms may explain the differences in the biological activities of Diocleinae lectins.

Comparative X-ray crystallographic analyses of dimannoside-complexed Diocleinae lectins isolated from *Canavalia maritima* and *Canavalia gladiata* showed that even very similar lectins differ in their interactions with disaccharides. This result suggests that differences in configuration and distance between the residues in the CRD can provide information on the ability of lectins to bind to glycoprotein and glycolipid receptors [31].

ConBr exhibits differences in the distances between specific amino acid residues that compose the primary carbohydrate binding site, thus altering the volume of the site [29]. Previous evidence indicates that these differences might explain the divergences in biological activities. The results reported in this study suggest that the lower volume found in ConBr could enhance the interaction with LPS, which are composed mainly by *O*-polysaccharides in *Rhizobium*.

**Figure 4:**
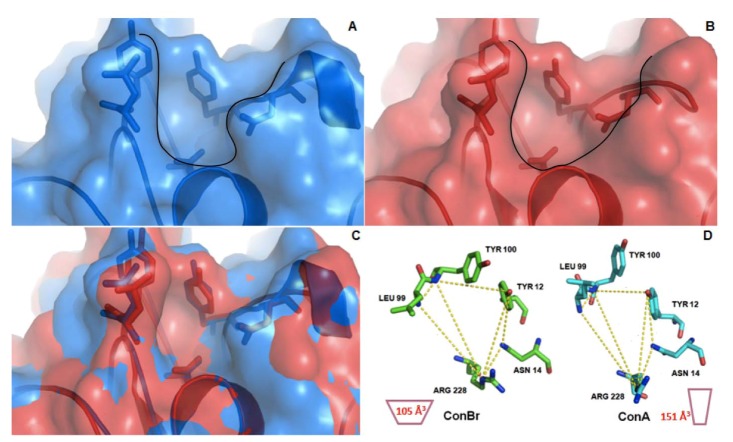
Surface analysis and structure alignment of ConA and ConBr tridimensional structures. (**A**) Carbohydrate binding site of ConA (Blue); (**B**) Carbohydrate binding site of ConBr (Red); (**C**) Structure alignment of ConA and ConBr showing differences in the topology which favors ConBr interactions with LPS; (**D**) Schematic representation of site volume in ConA (right) and ConBr (left). Calculated volume of each site is represented inside the schemes. Black lines in (**A**) and (**B**) delimitate the topology of sites.

Figure 4 highlights the differences in CRD design and volume between ConA and ConBr. ConBr has a more open carbohydrate binding site, whereas the site of ConA is narrower and deeper. Bezerra and colleagues [29] used this evidence to rationalize the stronger interactions between the *N*-glycans and both ConA and ConM *versus* the weaker interactions displayed by ConBr [29].

## 3. Experimental

### 3.1. Microorganism

*Rhizobium tropici* CIAT 899 was kindly provided by Embrapa Agropecuária Oeste, Dourados, MS, Brazil.

### 3.2. Lectin Purification

ConA and ConBr were obtained from the Biologically Active Molecules Laboratory (Biomol-Lab), Department of Biochemistry and Molecular Biology, Science Center, Federal University of Ceará, Brazil. The purification steps are described by Moreira and Cavada [32].

### 3.3. Lectin Complexation with Mannose

In bacterial growth experiments and with FITC-labeled lectins, ConBr and ConA were inhibited with D-mannose. The lectins were incubated with 0.1 M D-mannose at 37 °C for 30 min to induce complex formation. The solution was then filtering through a 0.22 µm Millipore filter and stored for later use.

### 3.4. FITC-Labeled Proteins

FITC-labeled proteins were prepared with inhibition buffer (0.1 M D-mannose in 0.1 M carbonate-bicarbonate buffer, pH 9.0), conjugation buffer (0.1 M carbonate-bicarbonate buffer, pH 9.0) and washing buffer (phosphate-buffered saline: 0.01 M sodium phosphate buffer, 0.027 M KCl and 0.15 M NaCl, pH 7.4). Initially, ConBr and ConA were dissolved in inhibition buffer and incubated at 37 °C for 1 h. Then, 250 µL fluorescein isothiocyanate (FITC) (500 µg/mL in conjugation buffer) was added drop wise. The solution was incubated for 2 h at room temperature with gentle stirring. Subsequently, unconjugated FITC was separated from FITC-lectin by molecular exclusion chromatography using a pre-equilibrated Sephadex G-25 column, and labeled lectins were eluted with washing buffer. The absorbances of all fractions were determined at 280 nm (protein) and 495 nm (FITC) to verify chromatographic efficiency. Labeled lectins were then dialyzed against 1 M acetic acid for 1 h to remove the inhibitor carbohydrate and extensively dialyzed against distilled water. Except for the inhibition with D-mannose, the same labeling protocol was used to label BSA with FITC.

### 3.5. Cultivation of Microorganisms

Strain CIAT899 was stock-cultured on yeast-mannitol (YM) [33] broth containing 20% glycerol and stored at −80 °C. The inoculum was then transferred to Petri dishes containing YM agar and incubated at 28 °C for 48 h. Subsequently, a colony was carefully transferred to 10 mL YM broth and incubated at 28 °C for 48 h under constant agitation. Immediately before use, the bacterial suspension was adjusted to 1 × 10^8^ cells/mL using a McFarland scale.

### 3.6. Lectin Fluorescence Microscopy

Bacterial cells were incubated separately with FITC, FITC-lectins, FITC-lectins in complex with D-mannose and FITC-bovine serum albumin (FITC-BSA) and agitated for 15 min. The cells were then washed 3 times in PBS, heat-fixed on slides and observed under a fluorescence microscope (Eclipse E200/epi-fluorescence, Nikon, Tokyo, Japan) equipped with a digital camera (DS-2Mv, Nikon, Tokyo, Japan). Images were acquired with NIS-Elements version 2.3 software (Nikon, Tokyo, Japan). Cells were observed using 100× oil-immersion objective with 1.5 s of exposure and 1× gain. Bacterial cells without treatment were observed to verify intrinsic fluorescence. The resolution of all acquired images was 5.0 Mpixels.

### 3.7. Bacterial Growth Assay

Assays were carried out using 96-well polystyrene plates. Following serial dilution from 500 to 15.6 µg/mL, the lectins were mixed with 100 µL bacteria suspension (1 × 10^8^ cells/mL) and incubated at 28 °C for 12 h. A 20 µL aliquot of bacterial suspension was then inoculated into 180 µL YM broth and incubated at 28 °C. The optical density at 620 nm (ABS 620nm) was measured at 12, 24, 36 and 48 h using a microplate reader (BioTrak II-Plate Reader). BSA and 0.15 M NaCl were used as protein and negative control, respectively. Lectin-D-mannose complexes were used in the bacterial growth assays. To prevent carbohydrates from interfering with metabolism, an additional negative control consisting of 0.1 M D-mannose was used. The bacterial growth assays were designed with 9 repetitions per group.

### 3.8. Statistical Analysis

The normality of the data within each experimental group was verified with the Shapiro-Wilk test, and the groups were compared pair wise with Student’s t test. The level of statistical significance was set at *p *< 0.01. All statistical analyses were performed with the software STATISTICA version 10.0 (Statsoft Inc., Tulsa, OK, USA).

### 3.9. Structure/Function Analysis

For the structural analysis, the crystal structures of ConA (PDB code: 1JBC) and ConBr (PDB code: 3JU9) were visualized using COOT [34] and PyMol [35]. Volume calculations were performed using the Q-SiteFinder program [36].

## 4. Conclusions

In this study, lectins from *Canavalia brasiliensis* (ConBr) and *Canavalia ensiformis* (ConA) bound to *Rhizobium tropici*, but only ConBr affected bacterial growth. This might be due to an extended and more opened carbohydrate binding site presented by ConBr which favors the interaction with *Rhizobium* LPS. Based on our results, ConBr may be a useful biotechnological tool in studies on the interaction between rhizobia and host plants.
